# Initial Presentation of Disseminated Coccidioidomycosis with Ocular Lesions

**DOI:** 10.1155/2020/1305193

**Published:** 2020-03-02

**Authors:** Edward J. Quinlan, Veenu Gill

**Affiliations:** ^1^Retinal Consultants of Arizona, 13943 N 91st Ave, Peoria, AZ 85831, USA; ^2^Infectious Disease Physician, Banner Thunderbird Medical Center, 5555 W. Thunderbird Road, Glendale, AZ 85306, USA

## Abstract

Ocular involvement with disseminated coccidiodal infection is rare. Even rarer is a patient presenting with symptomatic chorioretinitis first, followed weeks later by systemic symptoms of disseminated coccidioidomycosis. This highlights the need for physicians to have a heightened suspicion for testing for valley fever in patients living in endemic regions who present with ocular inflammation so that rapid and timely initiation of antifungal therapy may prevent loss of vision.

## 1. Background

Ocular involvement is a rare complication of disseminated coccidioidomycosis. Few case reports have been described in the literature where eye lesions with or without visual symptoms were found in patients already diagnosed with coccidioidal infection. We describe here the case of a patient who presented with ocular symptoms and was later diagnosed with disseminated coccidiodal infection potentially resulting in delayed treatment and permanent visual loss.

## 2. Case Report

A 40-year-old male, native of Arizona with a past medical history of type 2 diabetes mellitus, hypertension, and obesity developed decreased visual acuity of the left eye. This was sudden in onset and not preceded by trauma or insect bites. He went to see an ophthalmologist, and from there, was referred to a retina specialist for retinal eye exam. There were no associated systemic symptoms at the time. During the retinal exam, he was noted to have a pocket of fluid under the retina, and a diagnosis of central serous retinopathy was entertained. Since this is a self-limiting condition, the patient was counseled and asked to return after one month for a follow-up eye exam.

**Figure 1:**
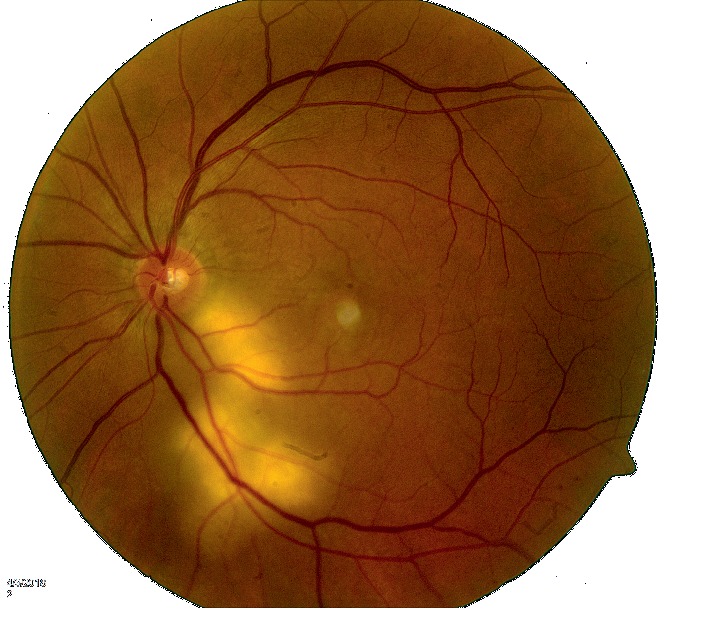
Active chorioretinitis extending inferotemporally from the optic disc of the left eye.

**Figure 2:**
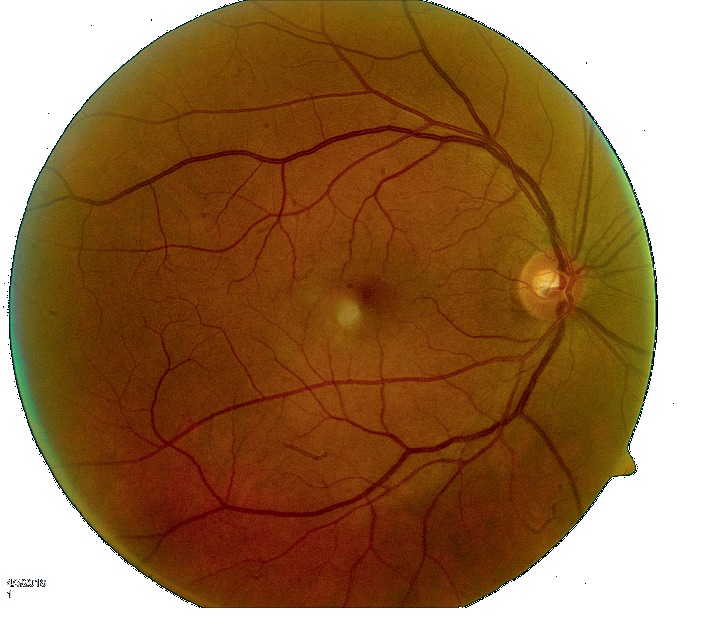
Right eye: normal exam.

Over the course of next few weeks, patient's visual symptoms worsened. He developed systemic symptoms of fevers, night sweats, fatigue, and generalized weakness. There was, however, no cough, chest pain, or shortness of breath. Initial blood work revealed positive IgM and IgG serology for coccidioidomycosis. He was started on oral fluconazole and referred to an infectious disease physician. Due to worsening visual symptoms, the patient went back to see his ophthalmologist. On exam, he was noted to have yellowish infiltrates beneath the retina on the left eye extending from the optic nerve to the retinal blood vessels. A diagnosis of chorioretinitis was made (Figures 1 and 2). Due to the recent diagnosis of valley fever, it was suspected that his eye involvement may be due to coccidioidomycosis. He was instructed to continue the oral antifungal medication and return for follow-up in a month.

**Figure 3:**
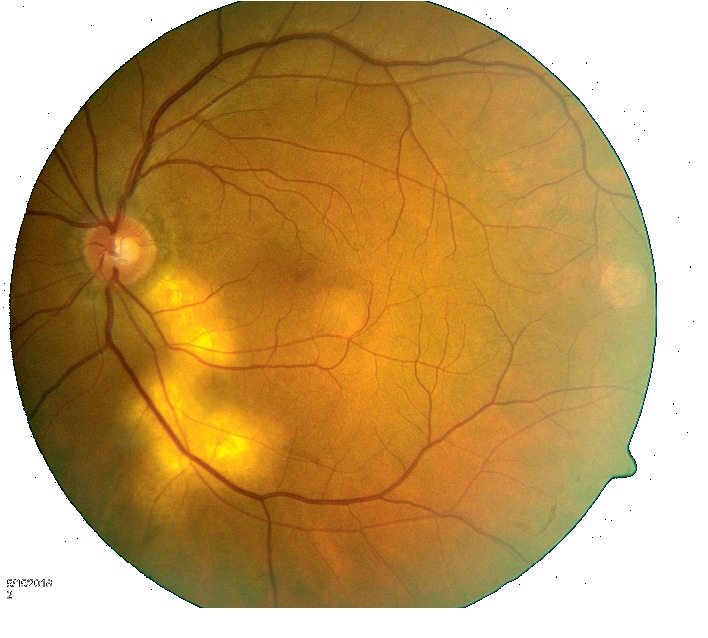
Chorioretinitis is seen to be regressing.

**Figure 4:**
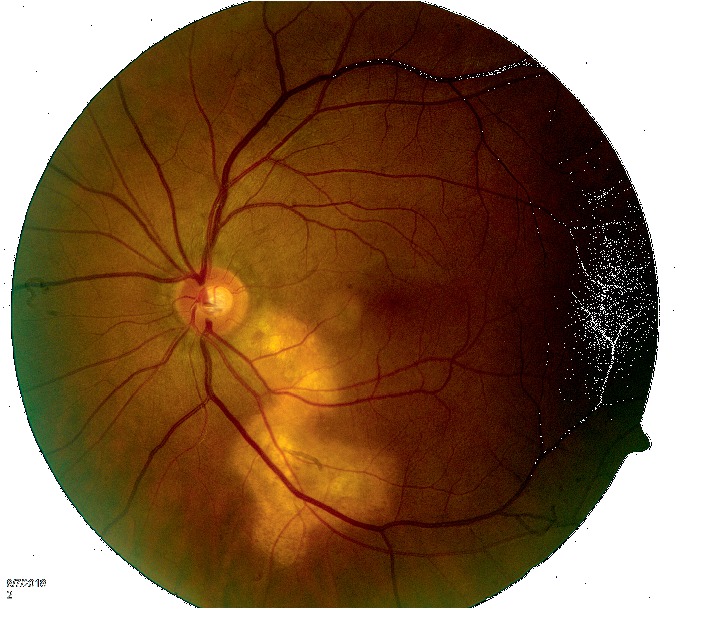
Chorioretinitis has completely regressed, and the patient is developing chorioretinal scarring.

At the initial diagnosis, his complement fixation titer was 1 : 8. He remained compliant with fluconazole. At follow-up visit with his ophthalmologist six weeks after starting antifungal therapy, the retinal inflammation had started to regress (Figure 3). At his 5 month follow-up visit, there was complete resolution of retinal inflammation with development of scarring (Figure 4). However, the patient had lost vision from the left eye. Despite resolution of the retinal inflammation, due to proximity to the optic nerve, there was irreversible damage to visual acuity. Over the next few weeks, the patient developed multiple scattered skin lesions, and his fluconazole dose was increased to 800 mg daily.

Unfortunately, repeat complement fixation titer worsened from 1 : 8 to 1 : 16 and then to 1 : 32 on subsequent labs at which point, he was switched to voriconazole 200 mg orally twice a day. He also underwent a spinal tap that was unremarkable. Coccidioides' antibody in the cerebrospinal fluid was negative. A CT scan of the chest showed bilateral pulmonary nodules. Interestingly, he did not have respiratory symptoms. After changing antifungal regimen, the patient had a slow but gradual clinical response with improving titers, resolution of fevers, night sweats, and skin lesions.

## 3. Discussion

Coccidioidomycosis is a fungal infection found mostly in the arid regions of the western hemisphere. Lungs are the primary portal of entry. Disseminated infection is known to involve the skin, bones including vertebrae and joints, and central nervous system. Ocular involvement with coccidioidomycosis is rare [1–3]. A few case reports have been described in the literature of conjunctivitis, iridocyclitis, chorioretinitis, and endophthalmitis [4–8]. However, ocular involvement in these cases was either subclinical or discovered incidentally in patients with a known diagnosis of valley fever or was the only evidence of disseminated infection in some patients [9]. Our case is unique as the patient presented solely with eye symptoms in the first few weeks without any systemic or pulmonary symptoms to suggest coccidioidal infection. It was, few weeks later, that he developed nonspecific complaints of fevers, night sweats, fatigue, and rash when he was tested and diagnosed with coccidioidomycosis. Even though his retinal inflammation resolved as a result of antifungal therapy, the damage to the optic nerve in the initial stages was permanent leading to irreversible vision loss. Unfortunately, he failed the standard treatment regimen and had to be switched to alternate drug therapy. Had he been diagnosed earlier, the visual damage could have been halted if not reversed. This highlights an important learning point for physicians to be aware of the possibility of eye involvement with disseminated coccidioidal infection as the initial presentation in endemic regions and to perform testing in a timely manner so that correct therapy can be initiated and permanent visual loss prevented.
